# Unleashing the Potential Role of CSR and Altruistic Values to Foster Pro-Environmental Behavior by Hotel Employees

**DOI:** 10.3390/ijerph182413327

**Published:** 2021-12-17

**Authors:** Jing Shao, Asif Mahmood, Heesup Han

**Affiliations:** 1 Business School, Qingdao University, Qingdao 266071, China; shaojing2208@hotmail.com; 2 Department of Business Studies, Namal Institute, Mianwali 42250, Pakistan; asif.mahmood@namal.edu.pk; 3 College of Hospitality and Tourism Management, Sejong University, 98 Gunja-Dong, Gwanjin-Gu, Seoul 143-747, Korea

**Keywords:** corporate social responsibility, pro-environmental behavior, altruistic values, hotel sector, environment

## Abstract

The hotel sector, around the globe, has a bad reputation due to its oversized carbon footprint. Therefore, this sector requires different approaches to improve its environmental management efforts. In this regard, the importance of employees’ pro-environmental behavior (PEB) has been recently discussed to reduce an enterprise’s carbon footprint. Reflecting this, the current work aims to improve PEB of employees as an outcome of corporate social responsibility (CSR) and altruistic values (ALV) in the hotel enterprises of an emerging economy. A total of 489 valid responses was collected from hotel employees, which were then analyzed through structural equation modeling (SEM). Different hypotheses were validated by employing SEM, and the results confirmed that CSR, directly and indirectly, via ALV as a mediator, improves PEB. The current work offers insights into the hotel industry for improving its environmental footprint through CSR and ALV. Moreover, academically, the current work advances the literature on CSR and environmental management from the perspective of hotel enterprises and by highlighting the role of individual values, especially ALV.

## 1. Introduction

Concern for the environment has emerged as one of the top priorities for society over the past few years. Undoubtedly, the environment is one of the most crucial elements for all stakeholders [1]. Given that many environment-related issues are rooted, at least partially, in an inappropriate interaction between humans and the environment, it is of utmost importance to promote pro-environmental behavior (PEB) among individuals at every level [2]. Several scholars have mentioned that issues related to the environment, including vulnerable climatic conditions, rising temperatures, droughts, floods, etc., can be significantly reduced if individuals at every level act in an environmentally friendly manner [3,4,5]. Despite the growing concern among scholars for PEB, a review of the literature unveils that most prior studies considered the outcomes of PEB. For example, the studies of Ahmad et al. [5] and Nisar et al. [6] indicated that PEB improves the environmental performance of enterprises. Likewise, Rajapaksa et al. [7] noted the significance of PEB for sustainable development. Instead of considering PEB’s outcomes, the authors believe that it is also important to explore the underlying mechanisms that encourages an individual to be engaged in certain environmentally specific behaviors. This perspective of PEB is documented in the literature from different perspectives. For example, the study by Byerly et al. [8] highlighted behavioral insights of individuals from the perspective of the environment. Similarly, Cinner [9] showed how cognitive biases and social influence could more effectively guide conservation behavior. The meta-analysis of Bergquist et al. [10] highlighted the importance of social norms to cause individuals to act in a pro-environmental way. From the perspective of consumers, Berger [11] employed signaling theory to propose that green products signal benefits to the environment, which ultimately shapes consumers’ purchasing behaviors so as to pay a premium for green products. However, considering the complex nature of individual behaviors, more research is required in the domain of PEB. Thus, one of the objectives of the current survey is to explore the underlying mechanisms that explain an individual’s engagement with PEB.

Quite recently, corporate social responsibility (CSR) has been recognized as influential in spurring PEB in individuals [12,13]. This perspective of CSR is a recent development, as the concept has long been related to different philanthropic-oriented activities. Arguably, this perspective is mainly an outcome of the burgeoning concern of individuals to protect the environment [14]. It is also established in the literature that the involvement of internal stakeholders, such as the employees of an enterprise, is critical for the effort to improve its environmental footprint [15,16]. As Zientara and Zamojska [17] noted, any enterprise that intends to reduce its carbon footprint within the CSR framework requires motivating its employees to be engaged in different environmental behaviors. This implies that, to address the environmental issues, an enterprise should embrace all hallmarks of a pervasive corporate philosophy, where every individual is engaged in sustainability initiatives [18].

Given that employees spend a significant amount of their daily time in workplaces, it is critical to promote eco-friendly behavior to achieve a better and sustainable future. Moreover, the employees of an enterprise have a profound knowledge of different operation processes, implying that their participation in sustainable initiatives can significantly improve the environmental footprint of an enterprise [17]. Yet, employees’ engagement in different extra-role behaviors (PEB in the current context) is attributed, at least in part, to different organizational factors. One such organizational factor that can urge employees to act pro-environmentally is the CSR orientation of an enterprise [19]. Therefore, another objective of the current analysis is to investigate the relationship between CSR and PEB.

**Figure 1:**
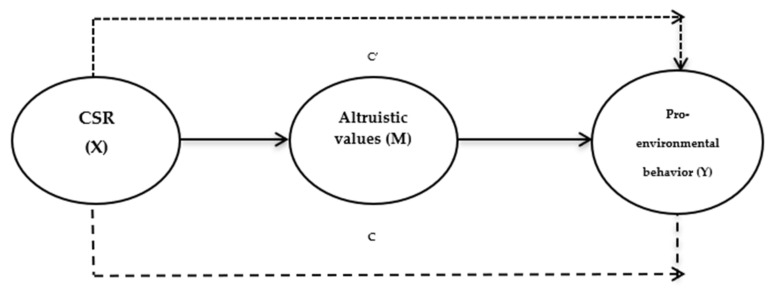
Research model for the current analysis: CSR (X) = the input variable, pro-environmental behavior (Y) = the outcome variable, altruistic values (M) = the intervening variable, C = the effect of X on Y in the absence of M, c′ = effect of X on Y in the presence of M.

Values are critical to influencing the behavior of individuals. According to Schwartz [20], values are trans-situational objectives that serve as guiding principles for individuals. Accordingly, values serve as building blocks of behavior for an individual by providing the basis for a specific action [21]. In the context of the environment, values, especially altruistic values, are associated with the collective welfare of society and the biosphere rather than seeking to serve the individual [22]. Stern and Dietz [23] pointed out that, from the perspective of environmental concern, values related to multiple targets (self or the biosphere) may guide an individual towards value-congruent information that eventually affects his attitudes and preferences, causing them to act pro-environmentally to protect the environment. The stable nature of individual values creates interest among theorists to study values more and more [24]. Given that altruistic values may predict individuals’ environmental beliefs, behaviors, and preferences, it is of prime importance to explore the importance of values in terms of the relationship between CSR and PEB. Hence, the current study introduces altruistic values as a mediator in the proposed relationship. Arguably the role of ALV in the proposed framework of the current survey provides a unique insight to spur the PEB of employees in the hotel sector of Pakistan. Given that both organizational factors and individual factors shape human behavior, the current research considers the CSR orientation of a hotel enterprise as an organizational factor, and ALV as an individual factor. Further, the proposed relationship was not well-explored under a unified model, especially in the context of the hotel sector, from the perspective of environmental management. The conceptual model of the current survey has been presented in Figure 1 to show the different hypothesized relationships.

Altogether, the current work attempts to answer the following questions:

(a) What is the impact of CSR perception of employees on PEB in the hospitality sector of Pakistan?
(b) What is the role of values, especially ALV, in fostering PEB of employees?
(c) Does ALV mediate between CSR and PEB?

The framework of the current analysis was tested in Pakistan, which is a Global South nation, and a recent victim of vulnerable climatic conditions. Of more importance, the environmental issues in the country have been increasing each year as the Environmental Performance Index (EPI) shows that Pakistan has performed poorly and is positioned in 176th place in the index [25]. Major environmental issues in the country include air pollution, deforestation, water scarcity, and the drying of wetlands. The country needs a war-like approach at every level to improve its environmental footprint. The current study has focused on the hotel sector of Pakistan to test the hypothesized relations (discussed above). The authors intentionally considered this sector due to the underlying reason that the hotel industry globally contributes almost 1% to the world’s total carbon emissions [26]. Given that hotel enterprises are identified as significant pollution contributors globally, the situation in Pakistan also reflects this. Hotels in Pakistan create different environmental problems related to the consumption of energy, water, and other natural resources. Moreover, this sector also contributes pollution through hazardous waste creation. It was identified in a recent report that this sector might improve its environmental footprint by promoting sustainable behavior by employees [27], which reflects the relevance of this sector to the theme of the current analysis.

Altogether, the current study intends to advance the available literature from three points of view. Firstly, the current study adds to the literature related to environmental studies and behavioral studies by presenting the CSR orientation of an enterprise as a potential motivator that engages employees to act pro-environmentally. Unlike the plethora of previous studies [28,29,30], the current study attempts to advance the field from the perspective of the environment. Secondly, the importance of values to influence the environmental behavior of individuals is pre-established in the literature [31,32]; such studies fail to note the mediating effect of values to spur PEB. Similarly, the relationship of altruistic values with CSR is an under-researched area. Thirdly, the bulk of the literature related to CSR and the environment has considered regions of the Global North, neglecting territories of the Global South. Given that CSR is culture and context specific, it is unwise to assume the findings from the Global North will produce the same results in the Global South. The remainder of the current work is composed of four parts. The next part deals with the theory and related literature to develop the hypotheses and research framework. Similarly, the methodology part discusses the sample, data collection procedures, and instrumentation. The last two parts are dedicated to the results and discussion, respectively. The results deal with the statistical analyses and outcomes, whereas the discussion addresses the study results in light of previous findings. This part also discusses the theoretical and practical implications of the current study.

## 2. Theory and Related Literature

The theoretical framework of the current analysis is underpinned by stewardship theory to develop the logic of the proposed relationship and to formulate the hypotheses. This theory was initially developed by Donaldson and Davis [33], and it contends that every human being is a steward and is assumed to work for the benefit of others. In an organizational context, this theory is advantageous to explain employee involvement in different extra-role engagements. More specifically, the concept of environmental stewardship is central to sustainability at every level of an organization. Stewardship theory better fits in the lexicon of CSR from the perspective of environmental improvement. For a long time, CSR scholars used stakeholder theory to explain individual behaviors in an organizational context [34,35,36]; however, the authors feel that this theory is slightly at odds with describing the full potential of CSR to address the environment. This viewpoint is rooted in the stakeholder theory, as this theory places social responsibility of a business on the direct stakeholders of an organization.

Given that environmental responsibility belongs to everyone, this theory is at odds with significantly improving environmental quality through CSR. This view is also supported in the work of Oliver [37] and Filipovic et al. [38]. Unlike the instrumental view of stakeholder theory for society and the environment, the stewardship theory opts for a normative approach that considers a wider approach to protect society and the environment, beyond the fiduciary obligations of an enterprise. This is why contemporary scholars have paid considerable attention to the stewardship theory so as to integrate it into corporate governance to improve the environment. The studies of Murtaza et al. [12] and Suganthi [39] are some recent contributions in this vein. 

It is generally established in the available literature that the CSR activities of an enterprise can significantly shape the behavior of employees [40,41]. Specifically, it is argued that, as the CSR commitment of a corporation is an extra-role commitment in the larger interest of society and the environment, it may trigger extra-role behavior from the employees [42,43]. Given that PEB is also an extra-role behavior of employees, it is logical to assume that the CSR engagement of an enterprise can significantly promote environment-specific behavior (an extra-role behavior) among employees. The current work also refers to the seminal work of Dewhurst et al. [44], who mentioned that CSR is less suitable to spur an employee’s bottom-line performance. Indeed, the concept is well placed to explain the engagement of employees in different additional roles. In the current context, when employees of an enterprise see socially responsible behavior to preserve nature and society, it infuses them with a sense of stewardship to act in the same way. Supposing that Rokeach [45] was right in stating that every person is concerned with caring for others, in that case, it is logical to assume that employees of a socially responsible organization can show concern for the protection of nature. At the same time, it was also reported that different factors influence individual value preferences. Specifically, it has been argued that, generally, prosocial value orientation exists among individuals. However, such value orientation is in tension with other values. For example, Ackermann et al. [46] stated that people were more willing to benefit people in comparison to environmental concerns. They also concluded that individuals could be more motivated for environmental issues if they are also informed that global warming is a real danger for humanity. Fehr and Fischbacher [47] also reported that altruism is critical to guide behavior, but that individual heterogeneity is also a matter of concern between altruism and selfishness. They also mentioned that a minority with high altruism could eventually pressure the selfish majority to cooperate, or vice versa. Thus, preference heterogeneity may also influence potential social preferences among individuals.

The scholars Glavas and Piderit [48] also predicted that employees are better engaged with an organization that shows caring values for others. Following stewardship theory, the workforce in a socially responsible enterprise holds their value of caring for others more strongly, urging them to practice behaviors, on their part, that can support the sustainability initiatives of the enterprise [49]. Specifically, employees perceive the CSR orientation of an enterprise as an act of steward towards society, which the employees also adopt. Observing this stewardship philosophy, employees are also expected to convert themselves into stewards to preserve nature. Additionally, Arnaud and Sekerka [50] argued that the moral values of an enterprise are pertinent to sustainability (CSR, for example) and could significantly shape the perception of employees and their environmental behaviors. They also mentioned that this involves a socialization process, that is, employees learn such shared values, which eventually prompt them to act pro-environmentally.

Similarly, Norton et al. [51] noted that the sustainability initiatives of an enterprise are assumed to permeate throughout the overall organizational climate, which has a definite impact on employees. Given that employees respond positively to the CSR initiatives of an enterprise, and also by referring to stewardship theory, it is logical to propose that CSR will infuse environmental values into employees, which urges them to then act pro-environmentally. Therefore, the following hypotheses are proposed:

**H1:**  *The CSR activities of an organization positively influence the PEB of employees.*

Though the concept of altruism is fundamentally assumed at the individual level, the authors align themselves with the standpoint of Elshuis [52], who argued the concept from an organizational perspective. Given that values are shared culturally, an organization’s socially responsible culture can significantly influence the moral values of employees relating to altruistic concern, including concern for society [53]. At the same time, employees with specific values could self-select into firms with specific cultures, as indicated in the studies of Bowles [54] and Burks et al. [55].

Assuming that values are central to an individual’s behavior while they are performing certain actions, it is possible to assume that there is moral value congruence between an employee and a socially responsible enterprise [17]. Although personal values determine individuals’ behaviors, they also take shape through the milieu in which they interact in a workplace. Moreover, social information processing theory [56] argues that the social environment (organizational environment in the current context) significantly predicts individual attitudes and behaviors, as the milieu enables an explanation of the construction of meanings via socially responsible beliefs and attitudes [57]. Berger [11] proposed a model of tipping pro-environmental norm diffusion, which suggested that the social preference of a population and the cost of adopting PEB can activate tipping for eco-friendly behavior. They also explicitly mentioned how ALV could promote PEB among individuals and how decision-makers could promote PEB diffusion, given specific levels of ALV in a population (like employees in the current case). In this vein, Berger [58] showed how social tipping interventions could guide sustainable behavior through self-reinforcing feedback. Their work also indicated that social tipping interventions with regular normative feedback could transform eco-friendly consumption from a minority behavior into a social norm.

Therefore, there is environmental value congruence between a socially responsible organization and the altruistic values of its employees. This viewpoint suggests that individuals’ social values converge with the CSR orientation of an enterprise, as supported by the work of Zientara and Zamojska [17]. In the given context, it is argued that employees with altruistic values are greatly influenced by the CSR engagement of an enterprise, as both the altruistic values of the employee and the CSR activities of enterprise stress caring for others. In sum, the CSR engagement of an enterprise creates an environmental value congruence between employees and the enterprise; therefore, the following is proposed:

**H2:**  *CSR engagement of an organization may positively influence the altruistic values of employees.*

It has been stated that values arguably guide individuals to act pro-environmentally [59,60]. Specifically, it is established that altruistic values are crucial to shaping the PEB of individuals [61]. Several researchers have made considerable attempts to explain value systems [59,62]; however, the field is dominated by the seminal work of Schwartz [20], who provided a comprehensive framework to explain the value structure. This value framework identifies four higher-level values, among which two are highly cited in the environmental literature, especially from the perspective of PEB. This includes self-enhancement and self-transcendence. Pinto et al. [63] identified self-enhancement values as being “person-oriented”, while self-transcendence relates to social orientation. It was established that socially-oriented values are better suited to explain the pro-social behavior of individuals [64]. This perspective of the value system is similar to altruistic values in which an individual considers social benefit while they makes a decision. Altruistic values include both social and biosphere dimensions, which have been found to positively explain the pro-social behavior of an individual [65]. Research shows that individuals with strong altruistic values have a strong orientation to act in an eco-friendly manner [66,67]. Importantly, values are assumed to hold a universal view, irrespective of different cultures, whereas it has been stated that individual attitudes change relative to changes in culture. Referring to this, Milfont et al. [68] concluded that values are a critical predictor of individual behavior, regardless of culture. Based on the above literary discussion, it is logical to propose that altruistic values positively relate to the PEB of employees as the altruistic orientation of employees urges them to care for others; therefore, they are expected to engage in activities that can protect or preserve nature and society. 

Equally important to mention here is that values are found to influence social behavior significantly; however, given that values provide broad bases to form a belief, they usually indirectly affect the behavior of individuals, rather than having a direct impact [69]. This is why several studies introduced values as moderators or mediators to explain individual behaviors. For instance, the work of Romani et al. [53] considered altruistic value as a moderator between CSR and advocacy behavior. Likewise, Zasuwa [70] tested the moderating role of altruistic value to shape consumer response to the socially responsible initiatives of a corporation. However, different studies also noted the importance of values as a mediator to shape an individual’s behavior. In this respect, the study of Hardy and Carlo [71] validated the mediating role of pro-social values in shaping the pro-social behavior of adolescents. Sree and Gunaseelan [72] acknowledged the mediating role of altruistic values on psychological well-being in the same way. The current analysis argues that the relationship between CSR and PEB is better explained if the altruistic value is introduced as a mediator in the proposed research model (Figure 1). As stated earlier, the CSR commitment of an enterprise creates a value congruence between employees and a corporation. In the context of environmental management, there is environmental value congruence between the CSR engagement of an enterprise and the environmental (altruistic) value of an employee. This process is well suited to explain PEB. Therefore, the following set of hypotheses is proposed:

**H3:**  *Altruistic values and PEB positively relate to each other.*

**H4:**  *Altruistic values mediate between CSR and PEB.*

## 3. Methodology

### 3.1. Population, Sample, and the Data Collection

The hotel sector in Pakistan was the target segment for the current analysis. This sector has been operating in the country since its independence and has provided considerable support to the economy. Despite the significant growth of this sector during recent years, the sector has also been blamed for engaging in activities that give rise to environmental pollution [73]. According to a report, the hospitality sector accounts for more than 7% of the contribution to the GDP of Pakistan. Moreover, this sector employs a massive workforce (more than 3.8 million people), with a net employment contribution of more than 6% [74]. With the inclusion of new players each year, this sector is expected to maintain its growth in the future. Moreover, the improved law and order situation in the country also contributes to growth in this sector. However, as this sector is globally considered a sector that directly or indirectly contributes to environmental issues, promoting sustainable behavior among employees in this sector is of great importance. Currently, this sector operates with local, national, and international players, including Avari, Marriot, Carlton, Regent, Hotel Mövenpick Karachi, Pearl Continental, and Ramada Plaza.

Metropolitan cities in the country, for example, Karachi, Islamabad, Lahore, Faisalabad, etc., are considered industrial hubs of Pakistan. As enterprises often arrange their business meetings, trainings, and seminars in hotels, the sector is relevant for industry. Given this relevance, such cities have a large umbrella of national and international hotels [75]. This research study considered three cities: Lahore, Islamabad, and Karachi. The logic for considering these three cities for the purpose of the current research is two-fold. First, as these cities hold the largest umbrella of multiple national and international hotels, considering these cities for the current analysis adequately addresses the issue of sample representativeness. More importantly, the second reason to consider these cities, especially Lahore and Karachi, is that both of these cities are victims of climate change. Specifically, Lahore was globally rated as the most polluted city in 2020 [76]. In the same vein, Karachi also has a bad reputation in terms of pollution. Given that these cities have populations of several million, and because of the alarming climatic situation, the logic to consider these cities is self-explanatory. Islamabad, though not considered a polluted city compared to the others, is the country’s capital city and almost all major players in the hotel sector have a presence in this city, making it impossible for the authors to exclude this city from the current survey.

The selection of different hotels to be considered relevant was achieved through considerable effort on the part of the authors to identify the hotels that were involved in different CSR activities. For instance, the authors searched the web pages of different hotels to verify if a hotel had a CSR presence on its website. Moreover, in some cases, the authors also confirmed the CSR engagement of a hotel through in-person visits. To this end, it was noticed that all upscale hotels were involved in some sort of CSR activities. After this identification, the authors made formal contact with different hotels to seek formal permission to support the authors in the process of data collection in the larger interest of academia and the hotel industry. The authors approached hotels that responded positively to address different administrative and procedural issues (schedule, timings, and dates for the survey). Such issues eventually enabled the authors to proceed with the data collection process.

The data collection instrument for the current analysis was an adapted questionnaire. Though the instrument was adapted from valid and reliable sources, before finalizing the instrument statements and making them publicly available to respondents of the survey, the instrument was assessed by the experts in the field (both academics and professionals) for its suitability to serve the purpose of the current survey. After receiving expert assessment, the authors provided this instrument to different respondents of the survey. This process of instrument validation is in line with the guidelines of prior researchers [77,78].

Moving forward in the process of data collection, the authors also ensured that ethical protocols were followed. For instance, all respondents were assured anonymity. Moreover, informed consent was also provided for each respondent. The authors also observed the ethical guidelines given in the Helsinki Declaration [79]. The sample included the perceptions of employees with managerial and non-managerial ranks. The authors randomly approached respondents. Employees from different departments, such as kitchen, maintenance, administration, and others, were invited to participate in the survey. The authors initially distributed 700 questionnaires among the staff of different hotels and received 489 valid responses. Thus, the response rate for the current survey remained close to 70%.

### 3.2. Measures 

As mentioned earlier, the authors considered an adapted instrument for the current survey. Given that a it was a pre-existing instrument and had pre-established validity and reliability [12,80,81], it was logical to consider such an instrument. The items to operationalize the constructs of CSR were adapted from the seminal work of Turker [82]. To the best of the authors’ knowledge, this scale is one of the most cited scales for recording employees’ CSR perceptions in different settings. For instance, in the hospitality sector, Raza et al. [83] considered this scale in recent work. Moreover, Tian and Robertson [84] also employed this same scale in the casino industry. A sample item from the scale was “My hotel participates in activities, which aim to protect and improve the quality of the natural environment”. A total of twelve items were used to operationalize CSR. The Cronbach alpha value (α) of this scale was 0.92. Likewise, an adapted scale to operationalize the construct of PEB from the study of Robertson and Barling [85] was employed by the authors. The scale consisted of seven-items with an α value = 0.88. A sample item was "I turn lights off when not in use". The respondents were asked to indicate their response on a seven-point Likert scale. Finally, the scale of altruistic values (ALV) was adapted from De Groot and Steg [86]. The studies of Lee et al. [22] and Kim and Stepchenkova [61] also employed this scale. For this scale, respondents were asked to rate the items in terms of importance. For instance, respondents were required to rank the importance of "as a guiding principle in my life, I consider pollution prevention". The scale ranged from 1 to 7 (not important to extremely important). Table 1 presents the demographics of the respondents.

## 4. Results 

### 4.1. Testing Common Method Variance

In many types of research, especially in the social sciences, the issue of common method variance (CMV) is gaining mounting importance among contemporary scholars. Specifically, a case in which data for all constructs are collected from a single source (as with the current survey) is more prone to suffer from CMV [87]. The existence of CMV in a dataset implies that the variation in responses for a survey is associated with an improper instrument (a biased instrument) rather than presenting the case of changes in respondents’ perceptions [88]. Indeed, the existence of CMV can lead an analyst towards a false internal consistency, implying that the results drawn from a dataset were contaminated by CMV and will not be able to represent the true explanation of an event. Hence, the authors decided to check the existence of this potential issue before proceeding further. In this regard, the authors first performed Harman’s single factor test and then a common latent factor test to detect CMV issues. However, the results of both tests revealed that the data of the current work showed no potential issues due to CMV.

### 4.2. Construct Evaluation: Factor Loadings, Validity, and the Reliability

The non-existence of CMV led the authors to advance the data analysis process to a further level. Hence, the authors performed several tests to evaluate the constructs of the current analysis. For instance, the authors assessed the factor loadings of all the constructs to see if there were any factors with under loading (λ < 0.5) or cross-loadings (Table 2). The results revealed that there were no issues with the factor loading of any item. Moreover, the authors also evaluated the convergent validity and composite reliability of each construct (CSR, PEB, and ALV) and found significant results.

Next, the authors performed correlation analyses to see the value and nature of the correlation between different pairs of constructs (Table 3). The outcomes of correlation analyses unveiled that all cases showed positive correlations, implying that all constructs were positively related to each other. As an example, one can see that the correlation (*r*) between CSR and ALV was 0.44, which was positive, showing that these two were positively correlated. Likewise, the authors also tested the divergent validity for each construct to ensure that the items of one construct were dissimilar from the items of the other constructs. To do so, the authors evaluated the square root value of AVE for each construct and then compared it with the correlation values. In this respect, to qualify the criterion of divergent validity, a construct under observation needs to produce a higher value of the square root of AVE compared to the correlation. As an example, the square root value of AVE for CSR was 0.81, which was larger than the correlation values (0.48, 0.44).

Further, the authors developed different alternate measurement models and compared them (Table 4) with the hypothesized model (mediated model). Such a comparison revealed that the hypothesized model of the current analyses best fit the data, as all model fit values were improved in the case of the hypothesized model (*χ*^2^ = 1436.510, *df* = 509, *χ*^2^/*df* = 2.82, RMSEA = 0.046, CFI = 0.94, NFI = 0.93).

### 4.3. Hypotheses Evaluation

Finally, the hypotheses of the current analyses were tested for possible acceptance or rejection by employing the structural equation modeling technique (SEM). For this purpose, the structural model was developed twice. First, the structural model was developed to record the direct influence of CSR on PEB without considering any mediators. The results of the direct effect model (Table 5) showed that H1, H2, and H3 were acceptable, as their beta values were positive and significant (*β*1, *β*2, and *β*3 were 0.42, 0.36, and 0.29, with *p <* 0.05). These statistical results are in line with the first hypothesis, implying that all the hypotheses had their statistical significance proven and were thus accepted.

Secondly, the structural model was redeveloped, but this time ALV was considered in the model as a mediator. The results for the mediated model are presented in Table 6 As per these results, ALV partially mediated CSR and ALV (*β4 =* 0.10, *p <* 0.05). These results statistically prove H4 and statistically acknowledge the mediating role of ALV.

## 5. Discussion

The current analyses were carried out to test the relationship between CSR and PEB in the hotel sector of Pakistan. To this end, the survey’s findings validated that the CSR perceptions of a hotel can create feelings of responsibility among employees. Especially in the context of the environment, CSR engagement of a hotel enterprise can shape sustainable behavior in its workforce. Theoretically, the CSR commitment of an enterprise is considered an extra-role commitment (a commitment that is not formally required), which is well placed to spur extra-role behavior (PEB) by employees, rather than fostering their bottom line (related to economic efficiency) performance [44]. Moreover, as the extra-role commitment of an employee is an entire volunteer activity, the formal obligations and responsibilities of the enterprise will be considered the least by employees to spur their extra-role commitment. A socially responsible hotel enterprise gives its employees a sense of caring for others. Following the norm of reciprocity [89], the employees receive this sense of caring from the hotel enterprise, and then reciprocate it positively by acting pro-environmentally. Such findings are in line with the findings of prior researchers [14,39,90].

Another important objective of the current analyses was to test the mediating role of altruistic values between CSR and PEB. In this respect, the statistical findings confirmed that the altruistic values of an employee provide added support to explain the CSR-PEB relationship. Altruistic values motivate them to act pro-environmentally, whereas the CSR orientation of a hotel enterprise further strengthens such caring-for-others values (altruism). On further note, Rokeach [45] argued that everyone has a personal value to care for others, and when employees perform their duties in a socially responsible enterprise, there is a value congruence between employees and the enterprise. However, as stated earlier, preference heterogeneity can significantly influence the potential of social preferences/values for promoting pro-environmental values. Perhaps this is why the mediation effect, though significant, was a bit low (23.8%). When applied to the current research, the environmental values of employees are in congruence with the CSR orientation of a hotel enterprise, which together develop a strong commitment from the employees. Thus, they not only perform their formal job obligations, but also proactively engage in different extra roles, including PEB. The role of altruistic value in spurring employee environmental behavior has been established in the existing literature [22,31]; however, the current study notes its mediating potential between CSR and PEB.

Lastly, the current study also proposes the stewardship theory as a more suitable theory than neighboring theories (for example, the stakeholder theory). The reason for the above argument was explained earlier. Given that theories like the stakeholder theory only focus on close stakeholders of an enterprise, such theories are not well-placed to explain the broader horizon of CSR for the environment. For example, considering better employment conditions for employees is an important CSR act of an organization; however, creating a positive impact on the general community and the environment has far-reaching consequences in the long term. The importance of stewardship theory from the perspective of the environment was, therefore, considered in some recent CSR studies [91,92].

### 5.1. Implications for Theory 

The current study advances the domains of CSR, employee behavior, and environment management in four ways. First, unlike the majority of previous studies [28,29,30], the current study discusses the potential role of CSR in addressing environmental problems in society. While the current perspective of CSR exists in the literature, most studies were conducted in the Global North [93,94], neglecting the Global South’s territories. Second, given that a plethora of CSR studies have been conducted in the context of the stakeholder theory, the current study advances the field by mentioning the limited ability of this theory to improve the environment. Thus, the current study considers stewardship theory as a more appropriate idea that considers the larger perspective of care for society and the environment. Third, most of the previous literature has focused on the outcomes of PEB; however, the underlying mechanism that keeps employees motivated to act pro-environmentally was neglected. The current study makes an effort to advance the literature by focusing on the underlying motivators of PEB rather than assuming its outcomes. Finally, the role of values, especially altruistic values, has been noted by different scholars as influencing the environmental behavior of employees [31,32]; however, such studies only noted the direct importance of such values in the context of their influence on PEB. The current study takes a different view to advance the field by proposing a mediating role by altruistic values between CSR and PEB.

### 5.2. Implications for Practice

Importantly, the current study offers some important practical implications, especially for hotel enterprises in Pakistan. The hotel sector of Pakistan should observe the encouraging results of this study to improve the environmental footprint of this sector through CSR. Currently, this sector largely focuses on the philanthropic dimension of CSR, which while also important, neglects the environmental role of CSR in a country that is already facing vulnerable climatic conditions, which is unwise. Some upstream hotels in Pakistan, for instance, Serena hotels and the Mövenpick hotel, have a dedicated commitment to CSR and have introduced different CSR projects to the general community (i.e., “Karighar” from the former and “Kilo of Kindness” from the latter), such programs only focus on the philanthropic importance of CSR. The hotel sector must reconsider its CSR philosophy by better incorporating the environmental dimension so that a better and sustainable future in the country is a viable option.

Moreover, hotel management is urged to communicate with its stakeholders, including the employees, that their hotel strongly considers the environment in their business strategies. The sector can observe the sustainable initiatives of hotel enterprises in the Global North, where clear CSR communication has significantly improved the environmental behavior of employees. To spur the moral values of employees, management in this sector can arrange different seminars and workshops with a special focus on employees to encourage environmental values. Finally, the negative environmental impacts of hotel enterprises must be mitigated if these enterprises desire to be sustainable in the future. In this respect, hotel management must not only improve their environmental footprint through different sustainability initiatives at an organizational level (e.g., green energy), but also through a better environmental behavior by its employees, for which a well-planned CSR strategy is a way forward. 

### 5.3. Limitations and Future Research Directions

Despite the current study discussing some critical implications for hotel enterprises in Pakistan, it also has some limitations. First of all, the study sample was limited to three cities, though these cities hold the largest share of the hotel business in the country. Still, it is suggested that considering other cities will be important for future studies. Second, the study only recorded perceptual measures of CSR. Though such perceptual measures are helpful in a plethora of studies, using an objective measure of such constructs in upcoming studies may generate more realistic outcomes. Similarly, the current study’s findings may remain limited in scope because a cross-sectional survey design was employed, limiting the causality of association among different constructs. In this regard, a better approach for future studies may be to incorporate a longitudinal data design. As CSR is contextually and culturally specific, the current survey findings may remain similar for similar cultures (India, Bangladesh, etc.). However, due care is necessary for different cultures before interpreting the current survey results. Lastly, this research may possibility include a social desirability bias. A more thorough research design is recommended for future research to avoid this issue.

## 6. Conclusions

Given that concern for the environment has presently become a main topic of debate, all sectors are realizing the importance of sustainability. In this respect, rethinking sustainable practices in the hotel sector is becoming a matter of prime importance, as this sector is known for its oversized carbon footprint. To this end, the current study is one of the limited contributions that highlight the importance of CSR in improving the environmental behavior of employees in a hotel. As this sector constitutes workforce of several million people in the country, improving sustainable behavior among employees is of utmost importance. In this regard, organizational factors (CSR) and personal values (altruism) are at the heart of influencing an employee’s behavior. The important takeaway of the current research is that the CSR activities of an enterprise create a value congruence between employees and the enterprise, which creates a strong bond on the part of employees and makes them want to show extra commitment to a socially responsible enterprise. Therefore, to have a sustainable future in this sector, undoubtedly, CSR is a way forward for hotel enterprises operating in Pakistan.

## Figures and Tables

**Table 1:** Demographic profiles of the sample.

Demographic	Frequency (*n* = 511)	%
**Gender**		
Male	273	55.83
Female	216	44.17
**Age in Years**		
18 to 25	56	11.45
26 to 30	92	18.81
31 to 35	162	33.12
36 to 40	88	17.99
Over 40	91	18.61
**Experience (Years)**		
1 to 3	76	15.54
4 to 6	184	37.63
7 to 9	167	34.15
10 or more	62	12.68
**Category**		
Manager/supervisor	136	27.81
Non-manager	353	72.19

**Table 2:** Factor loadings, convergent validity, and composite reliability.

Item	λ	λ^2^	E-Variance	∑λ^2^	Items	AVE	CR
My hotel participates in activities that aim to protect and improve the quality of the natural environment (CSR-1)	0.79	0.62	0.38				
My hotel makes investments to create a better life for future generations (CSR-2)	0.77	0.59	0.41				
My hotel implements special programs to minimize its negative impact on the natural environment (CSR-3)	0.86	0.74	0.26				
My hotel targets sustainable growth, which considers to the future generations (CSR-4)	0.82	0.67	0.33				
My hotel supports the non-governmental organizations that work in the problematic areas (CSR-5)	0.89	0.79	0.21				
My hotel contributes to the campaigns and projects that promote the well-being of society (CSR-6)	0.71	0.50	0.50				
My hotel encourages its employees to participate in voluntary activities (CSR-7)	0.85	0.72	0.28				
My hotel’s policies encourage the employees to develop their skills and careers (CSR-8)	0.83	0.69	0.31				
The management of this hotel is primarily concerned with the employees’ needs and wants (CSR-9)	0.87	0.76	0.24				
My hotel implements flexible policies to provide a good work environment and life balance for its employees (CSR-10)	0.72	0.52	0.48				
The managerial decisions related to the employees are usually fair (CSR-11)	0.75	0.56	0.44				
My hotel supports employees who want to acquire additional education (CSR-12)	0.89	0.79	0.21	7.97	12	0.66	0.96
I print double-sided whenever possible (PEB-1)	0.92	0.85	0.15				
I put compostable items in the compost bin (PEB-2)	0.81	0.66	0.34				
I bring reusable eating utensils to work (PEB-3)	0.79	0.62	0.38				
I put recyclable material (e.g. cans, paper, bottles, batteries) in the recycling bins (PEB-4)	0.83	0.69	0.31				
I turn lights off when not in use (PEB-5)	0.74	0.55	0.45				
I take part in environmentally friendly programs (PEB-6)	0.84	0.71	0.29				
I make suggestions about environmentally friendly practices to managers and/or environmental committees in an effort to increase my organization’s environmental performance (PEB-7)	0.86	0.74	0.24	5.57	7	0.80	0.94
Unity with nature (ALV-1)	0.71	0.50	0.50				
Preventing pollution (ALV-2)	0.82	0.67	0.33				
Protecting the environment (ALV-3)	0.70	0.49	0.51				
Respecting the Earth (ALV-4)	0.73	0.53	0.47				
Social justice (ALV-5)	0.88	0.77	0.23				
A world at peace (ALV-6)	0.94	0.88	0.12				
Helpful to others (ALV-7)	0.79	0.62	0.38				
Equality (ALV-8)	0.75	0.56	0.44	5.75	8	0.72	0.93

**Notes:** λ = Item loadings, CR = composite reliability, ∑λ^2^ = sum of square of item loadings, E-Variance = error variance.

**Table 3:** Correlation, discriminant validity, and model fit indices.

Construct	CSR	ALV	PEB
CSR	0.81	0.44 ^**^	0.48 ^**^
ALV		0.89	0.37 ^**^
PEB			0.85
Mean	5.62	5.85	6.11
SD	0.69	0.57	0.62

**Notes:** SD = standard deviation, ** = significant values of correlation, diagonal values = discriminant validity values.

**Table 4:** Model fit comparison, alternate vs. hypothesized models.

	Model-1	Model- 2	Model -3
*χ*2 (*df*)	1436.510 (509)	2108.731 (409)	1911.453 (518)
*χ*2/*df*	2.82	5.15	3.69
NFI	0.93	0.73	0.79
CFI	0.94	0.75	0.88
RMSEA	0.046	0.082	0.059

**Table 5:** The results for hypotheses (H1, H2, and H3).

Path	Relation	Estimates	SE	CR	*p-*Value	ULCI	LLCI	Decision
CSR→ PEB	+	(*β*1) 0.42 ^**^	0.038	11.05	***	0.293	0.216	Accepted
CSR →ALV	+	(*β2*) 0.36 ^**^	0.027	13.33	***	0.308	0.271	Accepted
ALV →PEB	+	(*β3*) 0.29 ^**^	0.025	11.60	***	0.246	0.208	Accepted

**Notes:** ULCI = upper limit confidence interval, LLCI = lower limit confidence interval, **, *** = significant values, + = positive relation.

**Table 6:** Mediation results for H4.

Path	Relation	Estimates	SE	Z-Score	*p-*Value	ULCI	LLCI	Decision
CSR →ALV→PEB	+	(*β4*) 0.10 ^**^	0.019	5.26	***	0.148	0.107	Accepted
Total effect		0.42						
Indirect effect		0.10						
Direct effect		0.32						
Proportion of mediation		23.8%						

**Notes:** ULCI = upper limit confidence interval, LLCI = lower limit confidence interval, **, *** = significant values, SE = standard error, + = positive relation.

## Data Availability

The dataset used in this research are available upon request from the corresponding author. The data are not publicly available due to restrictions i.e., privacy or ethical.
